# Expression of Concern: Rahman et al. An Overview of Antimicrobial Stewardship Optimization: The Use of Antibiotics in Humans and Animals to Prevent Resistance. *Antibiotics* 2022, *11,* 667

**DOI:** 10.3390/antibiotics13040353

**Published:** 2024-04-12

**Authors:** Antibiotics Editorial Office

**Affiliations:** MDPI, St. Alban-Anlage 66, 4052 Basel, Switzerland; antibiotics@mdpi.com

With this notice, the *Antibiotics* Editorial Office states its awareness of concerns relating to the originality of the concepts and analysis published in this review [1], cited below. Follow discussions with the authors, the Editor-in-Chief has decided to provide the authors with the opportunity to address these concerns and prepare a potential update to the publication, which will be considered by the Editor-in-Chief. Once this process has been completed, the Editorial Office will provide and update on this situation and announce any post-publication modification deemed to be necessary, as per MDPI’s Update to Published Papers policy.
